# Integrated maternal and child health services in Mozambique: structural health system limitations overshadow its effect on follow-up of HIV-exposed infants

**DOI:** 10.1186/1472-6963-13-207

**Published:** 2013-06-07

**Authors:** Diederike Geelhoed, Yves Lafort, Élder Chissale, Baltazar Candrinho, Olivier Degomme

**Affiliations:** 1 International Centre for Reproductive Health-Mozambique, Rua José Macamo 269 – 1A, Maputo, Mozambique; 2 Tete Provincial Health Directorate, Mozambique, B Filipe Manyanga, Tete, Mozambique; 3 International Centre for Reproductive Health, Ghent University Belgium, De Pintelaan 185 P3, Ghent, 9000, Belgium

**Keywords:** HIV-exposed infants, Mozambique, Integrated MCH care

## Abstract

**Background:**

The follow-up of HIV-exposed infants remains a public health challenge in many Sub-Saharan countries. Just as integrated antenatal and maternity services have contributed to improved care for HIV-positive pregnant women, so too could integrated care for mother and infant after birth improve follow-up of HIV-exposed infants. We present results of a study testing the viability of such integrated care, and its effects on follow-up of HIV-exposed infants, in Tete Province, Mozambique.

**Methods:**

Between April 2009 and September 2010, we conducted a mixed-method, intervention-control study in six rural public primary healthcare facilities, selected purposively for size and accessibility, with random allocation of three facilities each for intervention and control groups. The intervention consisted of a reorganization of services to provide one-stop, integrated care for mothers and their children under five years of age. We collected monthly routine facility statistics on prevention of mother-to-child HIV transmission (PMTCT), follow-up of HIV-exposed infants, and other mother and child health (MCH) activities for the six months before (January-June 2009) and 13 months after starting the intervention (July 2009-July 2010). Staff were interviewed at the start, after six months, and at the end of the study. Quantitative data were analysed using quasi-Poisson models for significant differences between the periods before and after intervention, between healthcare facilities in intervention and control groups, and for time trends. The coefficients for the effect of the period and the interaction effect of the intervention were calculated with their p-values. Thematic analysis of qualitative data was done manually.

**Results:**

One-stop, integrated care for mother and child was feasible in all participating healthcare facilities, and staff evaluated this service organisation positively. We observed in both study groups an improvement in follow-up of HIV-exposed infants (registration, follow-up visits, serological testing), but frequent absenteeism of staff and irregular supply of consumables interfered with healthcare facility performance for both intervention and control groups.

**Conclusions:**

Despite improvement in various aspects of the follow-up of HIV-exposed infants, we observed no improvement attributable to one-stop, integrated MCH care. Structural healthcare system limitations, such as staff absences and irregular supply of essential commodities, appear to overshadow its potential effects. Regular technical support and adequate basic working conditions are essential for improved performance in the follow-up of HIV-exposed infants in peripheral public healthcare facilities in Mozambique.

## Background

Despite recent rapid advances in the delivery of services for prevention of mother to child transmission of HIV (PMTCT) in many sub-Saharan African countries, the follow-up of HIV-exposed infants until the age of 18 months remains a critical challenge [1]. Adequate follow-up is important to ensure that HIV-exposed infants receive the necessary prophylaxis for PMTCT, are timely tested for possible HIV infection, and, if tested HIV positive, have access to treatment. Several studies have reported on barriers to follow-up in these infants, highlighting the importance of factors such as knowledge, practice and motivation of healthcare providers [2-4]. Furthermore, the way in which service delivery for the follow-up of HIV-exposed infants, including early infant diagnosis, is organised is also of importance, and in that context a better integration within other maternal and child health (MCH) services has been recommended [5,6]. However, to date little is known of its effects in practice.

In Mozambique progress has been made in PMTCT service delivery over recent years, even though the level of mother-to-child transmission of HIV was still estimated at 20% in 2011 [7]. Achieving adequate follow-up of HIV-exposed infants is among the main challenges of the PMTCT programme [7]. MCH service delivery in Mozambique is commonly organised around specific activities, and MCH nurses in public primary healthcare facilities usually attend their patients in a set order during the day: first new-borns, then pregnant women, then under-fives, and lastly, women attending for contraception. Follow-up of HIV-exposed infants is not routinely incorporated into any of these consultations, but rather provided in a specific ‘high-risk child clinic’, which may operate only on certain days of the week, or after or in-between other consultations. Therefore, it is possible that a considerable number of opportunities for the follow-up of HIV-exposed infants and for the provision of other MCH services are missed.

There is some evidence that the integration of PMTCT into routine antenatal and maternity care has had positive effects upon the care provided to HIV-positive pregnant women [1,8-11]. Along similar lines, the integration of care for mother and child into a one-stop, integrated consultation might have the potential to improve the follow-up of HIV-exposed infants by reducing missed opportunities for care. This study evaluated the viability of such one-stop, integrated care, its acceptability to healthcare providers, and its effect upon the follow-up of HIV-exposed infants and other MCH services, in the public healthcare system of Tete Province in Mozambique.

## Methods

Between April 2009 and September 2010, we conducted a mixed-method, intervention- control study in six rural public primary healthcare facilities, which were purposively selected in five districts of Tete Province, in north-western Mozambique, on the basis of their size and accessibility from the provincial capital. The aim of the study was to assess the viability and effect of integrated MCH care. Three healthcare facilities were randomly assigned (with coin toss) to the intervention, and three to the control group. The six facilities comprised of four smaller and two larger primary health centres at district or sub-district level, employing one to four MCH nurses each, and serving target populations of between 9,000 and 32,000 people. All facilities provide a similar range of MCH services, including care for normal childbirth, though only two offer basic emergency obstetric care. All six facilities have been providing PMTCT, including follow-up of HIV-exposed infants and infant diagnosis, for several years prior to participating in the study, though only one facility offered PCR testing at the time of our study.

The intervention consisted of a reorganization of MCH services, to deliver integrated, one-stop, consultations for mothers and their children up to 5 years of age. After appropriate training on how to integrate the services during the one-stop consultations, MCH nurses in the three healthcare facilities of the intervention group provided all recommended health interventions applicable to both mother and child, including follow-up of HIV-exposed infants and early infant diagnosis of HIV, during the antenatal, postnatal, family planning, growth monitoring, high-risk child and vaccination consultations. In the healthcare facilities of the control group, the same services were provided separately, one type of services after another, as is routine in the Mozambican public healthcare system. Care was taken not to create an artificially ideal situation with respect to availability of staff or supply of medication and other consumables, but to implement the study within the day-to-day practice of peripheral public primary healthcare facilities, in order to be able to assess the effect of integrated care in a real-life setting.

### Qualitative methods

To explore perceptions and experiences of healthcare providers in the six participating facilities with the one-stop, integrated MCH consultations and general MCH service delivery, they were interviewed at the start, after six months, and at the end of the study, using semi-structured questionnaires developed for each stage of the study. Topics discussed during these conversations included: general work satisfaction; satisfaction with the one-stop consultations (in the intervention group only); satisfaction with data registration tools; perceived situation regarding workload; situation regarding general health care facility conditions including availability of staff and commodities; perceived challenges for appropriate service delivery; and suggestions for improvements. All interviews were recorded and transcribed in Microsoft Word, with the aid of notes taken during the conversations. This qualitative information was manually analysed for themes and differences between the intervention and control groups.

### Quantitative methods

The clinical data on PMTCT, follow-up of HIV-exposed infants, and other MCH activities that had been routinely collected during the six months period before the intervention were used as baseline. During the 13 months of the intervention, the same data were collated during monthly supervisory visits to all six healthcare facilities. A list of collected variables is presented in Table 1. To increase the quality of routinely collected data and to allow for additional details, all participating healthcare facilities recorded their activities during the intervention period in an integrated registration book developed for the study. In addition, during the supervisory visits in all participating healthcare facilities technical assistance on MCH activities was provided, and information regarding presence of health personnel and availability of drugs and testing commodities was recorded. The monthly numbers of clients attended at each MCH service were entered in Microsoft Excel, and analysed with Stata/IC 11.0 (StataCorp LP, College Station, TX, USA) and R version 2.13.0 (The R Foundation for Statistical Computing) for significant differences between the periods before and after the intervention, between the healthcare facilities in the intervention and control groups, and for time trends. A generalized Poisson model allowing for overdispersion was used, controlling for a possible cluster effect. Pre-post differences and effect sizes were calculated with their p-values.

**Table 1:** List of variables from MCH activities collected monthly in the participant health care facilities

**Maternal health**	**Child health**
1 Number of pregnant women diagnosed with HIV in antenatal care	1 Number of live newborns from HIV-positive women giving birth in the health care facility (or at home but presented within 48h after birth)
2 Number of HIV-positive pregnant women starting antiretroviral prophylaxis or treatment in antenatal care	2 Number of live newborns from HIV-positive women giving birth in the health care facility (or at home but presented within 48h after birth) who received antiretroviral prophylaxis
3 Number of HIV-positive women giving birth in the health care facility	3 Number of HIV-exposed infants registered in the follow-up programme (per age group)
4 Number of HIV-positive women giving birth in the health care facility who received antiretroviral prophylaxis or treatment at childbirth	4 Number of HIV-exposed infants who visited for follow-up (per sex, age group and nº of times visited)
5 Number of childbirths of HIV-positive women with antiretroviral prophylaxis (or treatment) of both mother and newborn	5 Number of HIV-exposed children tested for HIV (per sex, age group and type of test used)
6 Number of women starting a modern contraceptive method (general and HIV-positive)	6 Number of HIV-exposed children tested for HIV (per sex, age group and type of test used) who tested HIV-positive
7 Number of women attended for post partum care (general and HIV-positive)	7 Number of children vaccinated (per vaccine: BCG, DPT3, Measles)
8 Number of women of reproductive age supplemented with Vitamin A	8 Number of children supplemented with Vitamin A
9 Number of women of reproductive age vaccinated for tetanus	9 Number of children de-wormed

The study adhered to national standards for ethical research in health and was authorised by national and local health authorities. Ethical clearance was obtained from the Mozambican Ministry of Health and the Ghent University Hospital.

## Results

### Qualitative results

Overall, the delivery of integrated, one-stop, consultations for mother and child was perceived as feasible in all intervention health facilities, despite some structural difficulties in one facility. In view of the larger patient flow in this health centre, integrated MCH consultations were provided by two MCH nurses simultaneously, who both needed to have easy access to various facility-based clinical record books, in particular those for the follow-up of HIV-exposed infants. As individual clinical files or a records office were non-existent, it was deemed most practical for both staff to work in the same space which also contained the necessary clinical record books, attending two clients simultaneously, separated by a screen to protect their privacy. With this adjustment, staff at all intervention facilities readily implemented the integrated MCH care appropriately, after a short period of settling in the new routine, and continued to do so during the entire study period.

All personnel involved evaluated the integrated service delivery model positively. To them it made much more sense to attend a mother with her child in a single, one-stop consultation, than to attend the child first, and have the mother return later that same day to the same consultation room for her own attendance. In addition, they felt that the provision of the one-stop consultation with its integrated data collection book resulted in a more efficient service delivery, with less time spent on administrative tasks.

‘The introduction of this programme was very good, as before we did not manage to collect many data and to test many mothers! Before we did not manage, […] we did not test the mothers or their children, but now, with this organisation, we do test the mothers and their children. It was very good to introduce this programme’.

Participant 2, intervention group

None of the participating staff reported a lack of technical knowledge or motivation as a factor impeding their daily work, though all appreciated the regular supervisory visits during the study period. In their view, these visits provided them with an opportunity to clarify doubts and receive updated information, and they highly valued the regular interest in their work and conditions.

‘Regarding the visits, these are an incentive for us. They make … that we feel a bit better, we feel better when we receive visits. It means that the efforts we make are important, that we do important work. So when we receive visits, it motivates us, it improves our morale’.

Participant 3, intervention group

According to the staff, the most important factors restricting performance at all participating healthcare facilities were frequent staff absences, resulting in an excessive workload for the remaining nurse(s), and the irregular supply of essential consumables and drugs. This was confirmed during the supervisory visits. During the study period it was occasionally possible to remedy such shortages in dialogue with district and provincial health authorities.

‘Only yesterday they brought me HIV tests, and since the beginning of the month till now, how many women already came here? A great many! They always send me 100 tests, since I started to work here, 100 tests per month. Now, when I have 100 tests, I keep 10 for the delivery room, for those women who were not tested yet, at least they can be tested during labour. Some others, 10 or 20, I send to the general outpatients consultations. So what is left here for MCH is very little! Only about 80 women can be tested. The rest, nothing! It is not sufficient. We should receive more, because many cannot be tested! Because of a lack of tests!’

Participant 4, intervention group

*‘At this moment, since the beginning of the month, I work here alone. Alone, I have nobody to help me! So, this is how I do my work now: one day I work in MCH, vaccinations, family planning, the next day I do general outpatient consultations, because to do it all in the same day, that’s what I cannot manage anymore. The same when my colleague* [who was absent at the time of the interview] *remains here alone, she does not enter here in the MCH consultation room, she only does the general outpatient consultations … yes … it is a lot of work’.*

Participant 6, control group

### Quantitative results

Table 2 presents the results of the average monthly number of MCH consultations in intervention and control facilities, during the six months before and 13 months following the start of the intervention. No data were available on follow-up visits of HIV-exposed infants during the pre-intervention period and therefore only data of other MCH activities were compared. At the control facilities, MCH attendance was overall significantly lower in the post-intervention than in the pre-intervention period. A less marked reduction was noticed in the intervention group. The difference in reduction was however not statistically significant.

**Table 2:** Average number of monthly MCH consultations in intervention and control clinics, pre- and post-intervention

**Average monthly Nr.**	**Control clinics**		**Intervention clinics**		**p value pre-post difference (control clinics)**	**p value control-intervention difference**
	**Pre-intervention**	**Post-intervention**	**Pre-intervention**	**Post-intervention**		
Maternal health consultations*	785	525	748	501	0.034	0.535
Child health consultations**	1355	857	1048	956	0.009	0.194

*Comprises women initiating family planning, postnatal care visits, Vit A supplements in postpartum women and tetanus vaccination in women of reproductive age.

**Comprises BCG, DTP3 and measles vaccinations, Vit A supplements and mebendazole presumptive treatment in children.

Because a pre-post intervention comparison for follow-up visits of HIV-exposed infants was not possible, we assessed time trends in the post-intervention period. The monthly number of HIV-exposed infants initiating follow-up increased significantly at the intervention facilities in comparison to the control group, but the monthly number of follow-up visits and of children tested for HIV decreased in comparison to the control group (Table 3). These results did not change after controlling for the availability of MCH staff, HIV testing commodities and MCH drugs.

**Table 3:** Time trends in number of monthly HIV follow-up visits during the 13 months post-intervention period

	**N**	**Time coefficient (control clinics)**	**p-value**	**OR intervention-control**	**p-value**
HIV-exposed infants initiating follow-up visits	76	−0.023	0.104	1.15	0.015
Follow-up visits by HIV-exposed infants	76	0.121	0.038	0.96	0.262
HIV tests in HIV-exposed infants	76	0.052	0.305	0.96	0.362

The results of the analysis of the age at which HIV-exposed infants initiated follow-up visits, the number of follow-up visits they attended, and the age at which they were tested for HIV, are presented in Table 4, which shows that both control and intervention facilities improved their follow-up of HIV-exposed children. In all six healthcare facilities the proportion of children initiating follow-up soon after birth (during the first three months of age) increased significantly between the first six months after the start of the intervention, and the seven following months. The proportion of children starting follow-up very late (after six months of age) reduced significantly. The difference between control and intervention facilities was small and not significant. Also the proportion of children attending for their fifth or more visit and the proportion of children tested timely (before the 10^th^ month) increased, while the proportion of children attending for their first visit and the proportion of children tested after 12 months decreased in both control and intervention facilities. These differences were more marked in the healthcare facilities in the control group.

**Table 4:** Distribution of HIV-exposed infants with follow-up by age at registration and number of follow-up visits

	**Control**		**Intervention**		**p value pre-post difference (control clinics)**	**p value control-intervention difference**
	**Jul-Dec 2009**	**Jan-Jul 2010**	**Jul-Dec 2009**	**Jan-Jul 2010**		
	**N=105**	**N=87**	**N=81**	**N=103**		
*Age at start of follow-up visits*						
Unknown	6.7%	12.6%	0.0%	0.0%	*NA*	*NA*
0-3 months	50.5%	72.4%	50.6%	75.7%	<0.001	0.394
4-6 months	18.1%	4.6%	18.5%	13.6%	0.013	0.288
7-18 months	24.8%	10.3%	30.9%	10.7%	<0.001	0.741
*Number of follow-up visits*						
First visit	40.0%	19.5%	30.1%	33.9%	<0.001	0.011
Second visit	29.5%	21.9%	31.6%	22.4%	0.004	0.694
Third visit	13.7%	17.4%	16.9%	14.1%	0.655	0.557
Fourth visit	6.8%	13.2%	11.0%	8.6%	0.179	0.296
Fifth or sixth visit	5.3%	18.1%	8.1%	10.9%	<0.001	0.037
Seventh or more visit	4.7%	9.8%	2.2%	10.2%	<0.001	0.726
*Age at serologic HIV test*						
7-9 months	20.0%	48.6%	22.9%	25.0%	0.028	0.092
10-12 months	60.0%	35.1%	40.0%	50.0%	0.423	0.940
>=13 months	20.0%	16.2%	37.1%	25.0%	0.149	0.059

During the post-intervention period information was collected on absence of MCH providers, stock-outs of HIV-testing commodities and stock-outs of one or more MCH drugs (Table 5). Absence of MCH staff occurred during 11 of the 69 observed months (15.9%), stock-outs of HIV testing commodities during 36 of 70 months (51.4%) and stock-outs of MCH drugs during 33 of 69 months (47.8%). Months with stock-outs of HIV testing commodities had a lower average number of HIV tests performed in pregnant women and HIV-exposed infants, although these differences had a low statistical significance. Similarly, months with a stock-out of MCH drugs had a lower number of pregnant women and children receiving medication for PMTCT, but again not statistically significant. Months in which one or more staff had been absent had a significantly lower number of HIV tests performed in children and a substantially lower number of HIV-exposed infants initiating follow-up visits.

**Table 5:** Effect of absence of staff, HIV testing commodities and PMTCT drugs on monthly MCH consultations in all clinics, post-intervention

	**N**	**OR**	**P value**
*Absence of MCH staff*			
HIV-exposed infants starting follow-up visits	67	0.20	0.200
Any HIV tests in HIV-exposed infants	69	0.06	0.027
Serologic HIV tests in HIV-exposed infants	69	0.10	0.030
*Absence of testing supplies*			
Pregnant women detected as HIV+	70	0.48	0.166
Any HIV tests in HIV-exposed infants	70	0.90	0.812
Serologic HIV tests in HIV-exposed infants	70	0.56	0.201
*Absence of MCH drugs*			
HIV + pregnant women initiating PMTCT	69	0.56	0.463
HIV + women receiving PMTCT during childbirth	69	0.32	0.196
HIV-exposed infants receiving PMTCT at birth	69	0.32	0.193

## Discussion

The integration of mother and child health care into one-stop consultations was found to be feasible and acceptable to the personnel of the selected rural public healthcare facilities in Tete Province, Mozambique. Our study shows that MCH nurses in the participating healthcare facilities were very well capable of changing their daily practice to accommodate such integration, and that they felt that this streamlined their service delivery. Integration of routine data collection tools in addition to the integrated clinical service delivery enhanced this perception of increased efficiency, also in the healthcare facilities in the control group. However, the practice of registering all consultations for follow-up of HIV-exposed infants in the same facility-based registration books rather than in individual files or patient-held records, made the actual implementation of integrated MCH care more difficult in the healthcare facilities with a larger patient flow, where more than one nurse attends clients simultaneously. Due to limitations in the number of available attendance rooms in many health facilities in Mozambique, it is not uncommon that two patients are attended by two providers in the same room at the same time, separated by a curtain or a screen, but this is obviously in conflict with the patient’s right to privacy.

In our study we did not assess client satisfaction, due to the limited local capacity to rigorously implement more complex study designs. Indirectly, the participating MCH nurses reported not to have received any complaints from clients, while in one of the intervention facilities clients apparently were pleased with the more streamlined attendance. However, before even considering any widespread introduction of one-stop, integrated MCH consultations, it would be important to assess its acceptability to the women attending these services.

Although there was high satisfaction among healthcare providers with the one-stop approach and a subjective feeling of increased effectiveness, this was not supported by the monthly MCH services attendance statistics. Attendance at most services was lower in the post-intervention period in both control and intervention facilities. The reason for this reduction in attendance is not clear, and it might have occurred due to dynamics not measured in our study. Possibly some contributing factors, such as absence of personnel, were more frequent in the post-intervention period, but data from the pre-intervention period were unfortunately not available. Another possible explanation is a higher quality of data registration in the post-intervention period, related to the integrated registration book introduced with the study in the six participating health care facilities, combined with possible over-reporting in the pre-intervention period. Use of routine data for scientific evaluations always has its limitations, in view of the lack of control over the quality of registered data. At the time of the study the MCH program in Mozambique used a multitude of different registration tools, some in books and some on loose sheets, with an irregular supply and limited availability and archiving at facility level. The rather short period for which we could collate pre-intervention data for our study (six months) is very much related to this limitation, as too often information was not traceable for months further in the past.

No pre-intervention data were available on the number of HIV-exposed infants followed at the facilities, and we could only assess potential post-intervention time trends. There were clear positive changes over time during the post-intervention period in the age at which children started follow-up, the number of visits they attended, and the age at which they were (serologically) tested for HIV. These effects were similar in the control and intervention healthcare facilities and could therefore not be attributed to the one-stop approach. In fact, our data show that all participating facilities gradually improved their follow-up of HIV-exposed infants. The earlier enrolment appears to be due to the fact that MCH nurses started to take advantage of post-natal visits to enrol the baby in the follow-up program, or even enrolled it already at birth. The number of follow-up visits of HIV-exposed infants increased as these were more and more often combined with child health visits for growth monitoring and vaccinations. Similarly, such child health visits increasingly included HIV testing for infant diagnosis of HIV-exposed infants. It is likely that these changes in daily practice of MCH nurses in both control and intervention facilities were induced by the regular supervisory visits, during which all MCH personnel received coaching and mentoring to improve their service delivery skills, including PMTCT. These quantitative data confirm the subjective sense of improved performance gained from the qualitative information from the staff interviews.

During the post-intervention period we recorded any periods that the healthcare facilities were confronted with absences of staff or with stock-outs of essential HIV testing commodities or MCH drugs. These periods were frequent, with about half of the observed months presenting stock-outs, and one in every six months presenting staff absences. Months with stock-outs had a lower number of activities involving the absent commodities and months with absence of staff had a substantial lower number of activities concerning follow-up of HIV-exposed infants. We therefore believe that these factors, combined with the regular staff support, had a greater effect on the performance of MCH services than the manner of organisation of these services.

Regular constructive supervision, adequate staffing levels and sufficient supply of commodities form important components of decent working conditions for health workers, essential to improve health worker retention and quality of services [12-14]. Unfortunately, as in the facilities in our study, such conditions are lacking in many healthcare facilities in Sub Saharan Africa. There is a severe lack of staff including midwives in many African countries, including Mozambique, which would need to double its number of midwives to remedy shortages, in addition to strengthening skills of the existing workforce [10,15]. UNFPA in Mozambique recently reported a poor availability of drugs for contraception and obstetric care, especially in peripheral health facilities, with frequent stock-outs [16]. WHO has also highlighted the poor availability in the public sector of a basket of essential medicines in many regions, including in Africa [17]. Both reports recommend reinforcing the acquisition and distribution chain in general, and of medicine policies in particular. Our study suggests, both qualitatively and quantitatively, that these structural limitations indeed constrain peripheral healthcare providers in their provision of MCH services, to such an extent that an even marginal improvement of their working conditions through regular supervisory visits provided during the study, was associated with a general improvement of the follow-up of HIV-exposed infants. Any potential effect of the reorganisation, integrating MCH service delivery, could not be shown in our study in the face of such limitations. We recommend therefore that the improvement of the basic working conditions of peripheral MCH staff in public healthcare facilities in Mozambique and similar settings, including regular support and an adequate supply of commodities, might be a more effective way to improve the follow-up of HIV-exposed infants, before considering any reorganization of service delivery.

## Conclusion

Despite improvements in some aspects of the follow-up of HIV-exposed infants, no progress attributable to one-stop, integrated MCH services was observed during our study. Structural healthcare system limitations, such as staff absences and an irregular supply of essential commodities, appear to have a larger effect. Regular technical support and adequate basic working conditions form valuable motivators and are of critical importance for improved staff performance in the follow-up of HIV-exposed infants in peripheral public healthcare facilities in Mozambique.

## Competing interests

The authors declare that they have no competing interests.

## Authors’ contributions

DG conceived of the study, participated in its design and coordination, performed the qualitative analysis, and drafted the manuscript. YL participated in its design, participated in the statistical analysis and helped to draft the manuscript. EC participated in the implementation of the study, in the qualitative analysis, and helped to draft the manuscript. BC participated in the coordination of the study and in the qualitative analysis, and helped to draft the manuscript. OD performed the statistical analysis. All authors read and approved the final manuscript.

## Pre-publication history

The pre-publication history for this paper can be accessed here:

http://www.biomedcentral.com/1472-6963/13/207/prepub
